# Flexible Transparent Conductive Film Based on Random Networks of Silver Nanowires

**DOI:** 10.3390/mi9060295

**Published:** 2018-06-13

**Authors:** Hui Xie, Xing Yang, Dexi Du, Yuzhen Zhao, Yuehui Wang

**Affiliations:** 1 Department of Chemistry and Biology, University of Electronic Science and Technology of China Zhongshan Institute, Zhongshan 528402, China; huixiefly@126.com; 2 State Key Laboratory of Electronic Thin Films and Integrated Devices, School of Microelectronics and Solid-State Electronics, University of Electronic Science and Technology of China, Chengdu 610054, China; shirleywyh@126.com (X.Y.); dudexi_work@foxmail.com (D.D.); 3 Department of Materials Science and Engineering, Tsinghua University, Beijing 100084, China; zhaoyz@mail.tsinghua.edu.cn

**Keywords:** silver nanowires, flexible transparent film, polyethylene terephthalate, sheet resistance

## Abstract

We synthesized silver nanowires (AgNWs) with a mean diameter of about 120 nm and 20–70 μm in length using a polyol process. The flexible transparent conductive AgNWs films were prepared using the vacuum filtration-transferring process, in which random AgNWs networks were transferred to a polyethylene terephthalate (PET) substrate after being deposited on mixed cellulose esters (MCEs). Furthermore, the photoelectric and mechanical properties of the AgNWs films were studied. The scanning electron microscopy images show that the AgNWs randomly, uniformly distribute on the surface of the PET substrate, which indicates that the AgNWs structure was preserved well after the transfer process. The film with 81% transmittance at 550 nm and sheet resistance about 130 Ω·sq^−1^ can be obtained. It is sufficient to be used as a flexible transparent conductive film. However, the results of the bending test and tape test show that the adhesion of AgNWs and PET substrate is poor, because the sheet resistance of film increases during the bending test and tape test. The 0.06 W LED lamp with a series fixed on the surface of the AgNWs-PET electrode with conductive adhesive was luminous, and it was still luminous after bent.

## 1. Introduction

There is increasing demand for suitable flexible transparent conductive materials due to the emergence of flexible plastic devices and the scarcity of indium resources. In recent years, silver nanowires (AgNWs) utilized for fabricating flexible transparent conducting films (FTCFs) for flexible electronics and transparent heaters have attracted significant attention for their excellent mechanical, optical, thermal, and electrical properties [1,2,3,4,5,6,7,8]. However, critical issues still exist that need to be addressed for the large scale application of AgNWs networks electrodes: (1) the low adhesion between AgNWs and the bare substrate; (2) the large contact resistance across the silver wire-wire junction [9,10,11,12,13,14,15,16,17]. To further optimize the performance of AgNWs-based electrodes, scientists have made much progress at improving the performance of silver nanowires networks electrodes. The adhesion of AgNWs to the surface of the substrate can be enhanced using the adhesion layer between AgNWs and the substrate, and high-intensity pulsed light sintering and pressure [9,10]. In addition, the removal of the insulating layer covered on the surface of the nanowires, or pressure treatment or heat treatment, can improve contact resistance across the silver wire-wire junction [10,13,14].

Until now, many approaches used to prepare silver nanowires networks have been presented including vacuum filtration, the drop-cast method, Meyer rod coating, and the transfer-printing technique [18,19,20,21,22,23,24,25,26,27]. However, it is very difficult to achieve a high quality film with low sheet resistance, high optical transmittance, and good adhesion between the nanowires and substrate surface at the same time. Vacuum filtration method is a commonly-used process in film production, which affords films with some advantages such as surface uniformity, controllable thickness, and reproducibility. To fabricate the flexible transparent conductive film, AgNWs networks usually are transferred to a transparent substrate after being deposited on the mixed cellulose ester film; this constitutes the vacuum filtration-transferring process. Moreover, it is very easy to generate cracks and other adverse defects during the transferring process from mixed cellulose ester to polyethylene terephthalate (PET).

Our initial work was the fabrication of flexible transparent AgNWs conductive film with the mixed cellulose eater (MCE) as a substrate; we obtained a good conductive structure on the MCE film using the improved vacuum-filtrating method [28]. In this work, we further transfer AgNWs on the surface of MCE to polyethylene terephthalate (PET) after dissolving the MCE. The good transferred conductive structure was obtained under our experimental conditions, and the photoelectric and mechanical properties of the AgNWs films were discussed.

## 2. Materials and Methods 

Silver nitrate (AgNO_3_, ≥99.8%) was purchased from Guangdong Guanghua Chemical Reagent Co., Ltd. (Guangdong, China), poly(vinylprrolidone) (PVP, C_6_H_9_NO)_n_; K30, Mw ≈ 10,000, was purchased from Guoyao Group Chemical Reagent Co., Ltd. (Shanghai, China); ferric chloride (FeCl_3_·6H_2_O, ≥99.5%) was purchased from Chengdu Kelong chemical Co., Ltd. (Chengdu, China); and ethylene glycol (EG, (HOCH_2_)_2_, ≥99.7%) and ethanol absolute (CH_3_CH_2_OH, ≥99.7%) were purchased from Tianjin Yongda chemical Co., Ltd. (Tianjing, China). 125 µm-thick polyethylene terephthalate (PET) film was purchased from Shanghai Fei Xia Plastic Hardware Co., Ltd. (Shanghai, China). Water mixed cellulose esters membrane (MCE, Ф50, 0.4 μm) was purchased from Tianjin Jin Teng experimental equipment Co., Ltd. (Shanghai, China). Silver nanowires were synthesized in our laboratory. All the chemicals were used as received.

**Figure 1:**
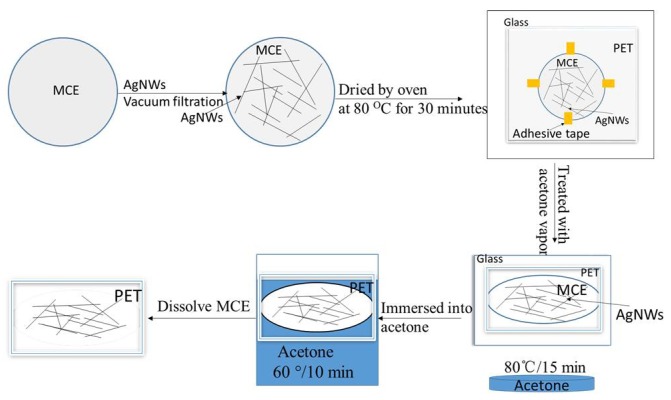
Schematic illustration of the preparing AgNWs-PET film process.

Silver nanowires with a mean diameter of about 120 nm and a length of 20–70 μm were synthesized by our reported polyol process [27]. 12.67 mg·mL^−1^ AgNWs suspensions were diluted down to 0.0022 mg·mL^−1^ with deionized water and sonicated for one minute. AgNWs diluent dispersions were dispersed into 500 mL deionized water and deposited on porous MCE membrane to form AgNWs networks with different densities by vacuum filtration. The AgNWs-MCE film was placed on a hard plastic plate with binder clips and dried by oven at 80 °C for 30 min. Further, the AgNWs-MCE film was fixed with four pins located in the middle of each boundary on a PET by adhesive tapes to ensure the MCE entirely was completely in contact with the PET. The AgNWs-PET-MCE film was treated with acetone vapor at 80 °C for 15 min. Then, the sample was immersed into acetone at 60 °C for 10 min to completely dissolve the MCE. Figure 1 shows the schematic illustration of the preparing AgNWs film process.

All scanning electron microscopy (SEM) images were taken on a FE-SEM Field emission scanning electron microscopy, S3400 N, Hitachi Co., Tokyo, Japan). The sheet resistance of the FTCFs was measured using a ST2263 four-point probe instrument (Suzhou Jingge Co., Ltd., Suzhou, China). Optical transmittance (Ts) spectrum was measured using ultraviolet-visible light detector (UV-1800 SHIMADZU Co., Kyoto, Japan). The phase structures were determined by X-ray diffraction (XRD) (Rigaku 2500 X-Ray DIFFRRACTOMETER, Rigaku Co., Japan ) on a scintage diffractometer with Cuk_α1_ radiation (λ = 1.54060 Å) at a scanning rate of 2°/min in the 2θ range from 20 to 90°. 3 M tape with finger pressure as a method of mechanical tape test is adopted to evaluate AgNWs adhesion property to the substrate. The adhesion performance of the AgNWs to the PET substrate is characterized through the sheet resistance changes. The sheet resistance is measured after 3 M tape was stripped off from the AgNWs film each 5 times. The bending test was carried out with lab-made apparatus with software recording film resistance and cycle number.

## 3. Results

**Figure 2:**
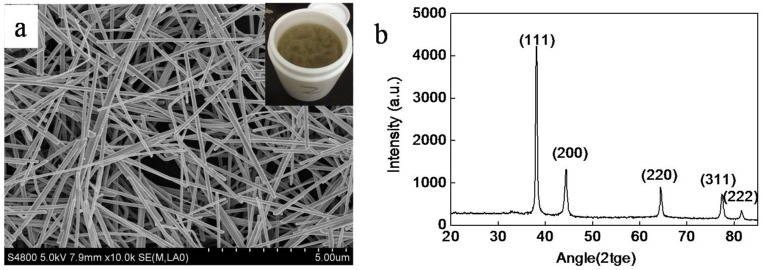
(**a**) SEM image and (**b**) XRD of the silver nanowires. The photo of silver nanowires synthesized by a solvothermal process is inserted in Figure 2a.

SEM image (Figure 2a) and XRD (Figure 2b) of the synthesized AgNWs are shown in Figure 2. A photo of the synthesized AgNWs by a solvothermal process is inserted in Figure 2a. Seen from Figure 2a, the range of diameter of AgNWs is about 120 nm, and the range of the length is about 20–70 μm. The color of the synthesized AgNWs solution is gray. Figure 2b shows five diffraction peaks, indexed to the (111), (200), (220), (311), and (222) planes of pure face-centered-cubic (fcc) silver crystals.

**Figure 3:**
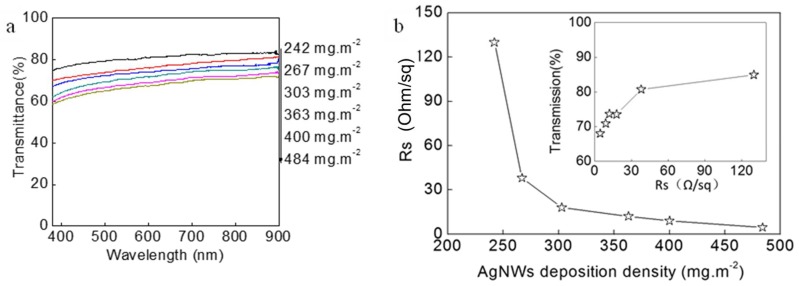
Relationship of optical transmittance spectra (**a**) and the sheet resistance (**b**) of AgNWs films versus the silver nanowires deposition densities of AgNWs-PET films.

The optical transmittance spectra (Figure 3a) and the sheet resistance (Rs) of AgNWs films with different deposition densities (in grams deposited per square meter) of AgNWs are shown in Figure 3. The relationship of the Rs and the transmittance at 550 nm of AgNWs film is inserted in Figure 3b. The transmittance was measured with a transparent PET film as the reference. Both of the transmittance and the Rs of the AgNWs films decrease with the increase of deposition density of AgNWs. When the deposition density of AgNWs is 242 mg·m^−2^, the transmittance of AgNWs film at 550 nm is over 84%, and the Rs is above 130 Ω·sq^−1^. Increasing the deposition density of AgNWs to 267 mg·m^−2^, a dramatic decrease in the Rs (38 Ω·sq^−1^) was observed in Figure 3b, and the transmittance of AgNWs film at 550 nm is 81% (Figure 3a), which is better applied to the transparent conductive film. The Rs and the transmittance of AgNWs film with 400 mg·m^−2^ deposition density decreases to 9 Ω·sq^−1^ and 71%, respectively. It is clear that the conductive networks of AgNWs gradually increase with the increase of the deposition density of AgNWs. Under the condition of the low deposition density of AgNWs, the increase of a small amount of AgNWs can improve the conductivity significantly. However, when the deposition density of AgNWs reaches a certain value, effective conductive networks form, which has a small effect on the deposition density of AgNWs with regard to conductivity. The dense conductive networks lead to a decrease in transmittance of film. 

**Figure 4:**
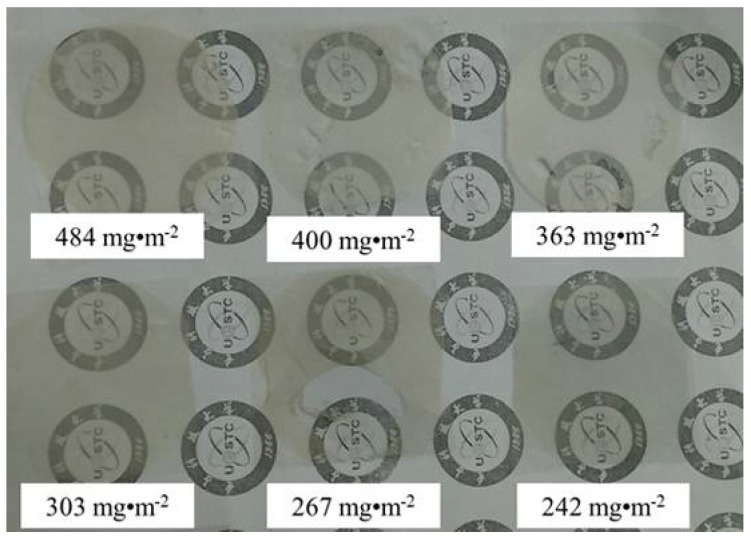
Photos of AgNWs-PET films with different silver nanowires volumes.

The photos of AgNWs films with different deposition densities are shown in Figure 4. Seen from Figure 4, the transparency of AgNWs films decrease with increasing deposition densities of AgNWs. However, the distribution of AgNWs transferred to the PET substrate is uniform.

**Figure 5:**
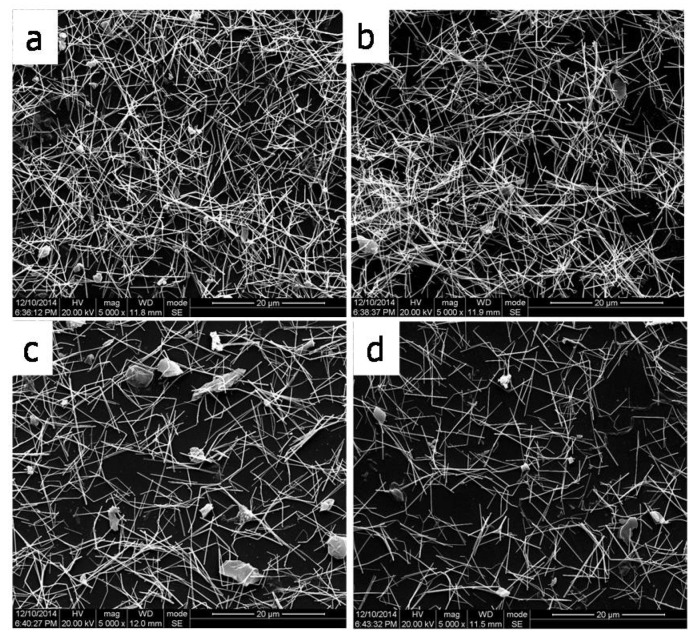
SEM images of AgNWs-PET films with different deposition densities of AgNWs, (**a**) 363, (**b**) 303, (**c**) 267, and (**d**) 242 mg·m^−2^.

Figure 5 shows SEM images of the AgNW-PET films with 363 (Figure 5a), 303 (Figure 5b), 267 (Figure 5c), and 242 mg·m^−2^ (Figure 5d) AgNWs, respectively. Seen from Figure 5, AgNWs covered on the surface of the PET substrate to form conductive network by crosslinking. The distribution of AgNWs transferred to the PET substrate is uniform. This is consistent with the phenomenon shown in the Figure 4. In this work, the sample-treated temperature with acetone vapor at 80 °C, and the sample-fixed with four pins located in the middle of each boundary on a PET were used. The highly treated temperature shortened the melting time of MEC, and the fixed sample reduced the deformation of the sample, thus preventing the accumulation of silver nanowires. Photos of silver nanowires films can be seen in Figure 5. The silver nanowires are evenly coated on the surface of PET, indicating that the transfer process is good. With the increase of AgNWs’ deposition density, more and more conductive networks are formed. 

**Figure 6:**
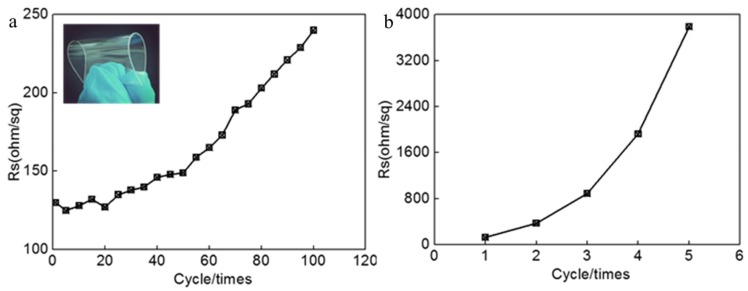
Rs of AgNWs films versus cycle time of folding up (**a**) and tape test (**b**).

Figure 6 shows the relationship of Rs of AgNWs film (the deposition density of 267 mg m^−2^) to cycles of folding up (Figure 6a) and tape test for 5 times (Figure 6b). The inserted is a photo of folding up. Seen from Figure 6, with the increase of the cycle member of folding up, the Rs gradually increases. After the 50 cycles of folding up, the Rs changed linearly depending on cycle members. Rs of AgNWs film increased nearly 2 times at 100 cycles of folding up. It is clear that after many cycles of folding up, some of the AgNWs came off the surface of the PET substrate because of the poor adhesion of Ag NWs and PET substrate, so the Rs decreased gradually due to the decrease of the conductive paths. We did not obtain the sheet resistance of film after tape test was repeated 6 times. It is clear that the Rs gradually decreases after tape test. We observed the AgNWs were removed from the PET substrate and adhered to the tape after each tape test, indicating that the adhesion of the AgNWs to the PET substrate is weak. 

**Figure 7:**
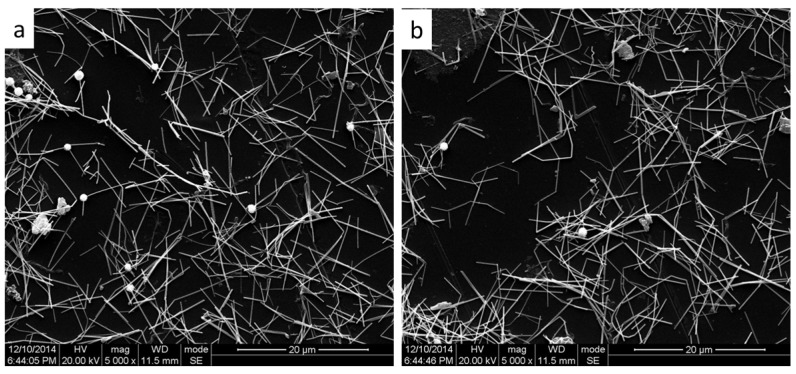
SEM images of samples after folding up (**a**) and tape test (**b**).

Figure 7 shows the SEM images of samples after folding up (Figure 7a) and tape test (Figure 7b). Compared with Figure 5c, it is clear that the deposition density of AgNWs on the surface of PET deceases after the cycles of folding up and tape test, indicating that the AgNWs fell off from the PET.

**Figure 8:**
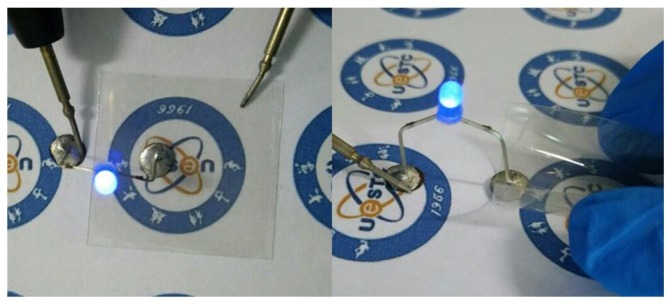
Photos of LED lamp device.

One 0.06 W LED lamp with series fixed on the surface of AgNWs-PET electrode with conductive adhesive were luminous, and they were still luminous after bent (Figure 8). 

## 4. Conclusions

Silver nanowires with a mean diameter of about 120 nm and 20–70 μm in length were synthesized using a polyol process. Further, the flexible transparent conductive AgNWs films with PET as a substrate were prepared using the vacuum filtration-transferring process. The results show that the AgNWs randomly and uniformly distribute on the surface of the PET substrate, which indicates that the AgNWs structure is preserved well after the transfer process. The film with 81% of the transmittance at 550 nm and 130 Ω·sq^−1^ sheet resistance can be obtained when the deposition density of AgNWs is 242 mg·m^−2^. It is sufficient to be used as a flexible transparent conductive film. The bending test indicated that the Rs gradually increases with the increase of the cycle member of folding up. After the 50 cycles of folding up, the Rs changed linearly depending on cycle members. The tape test indicated that the Rs gradually decreases after the tape test and the sheet resistance of film did not obtained after the tape test was repeated 6 times, indicating that the adhesion of the AgNWs to the PET substrate is poor. The 0.06 W LED lamp with series fixed on the surface of AgNWs-PET electrode with conductive adhesive was luminous, and it was still luminous after being bent.

## References

[1] Jo W., Kang H.-S., Choi J., Lee H., Kim H.-T. (2017). Plasticized polymer interlayer for low-temperature fabrication of a high-quality silver nanowire-based flexible transparent and conductive film. ACS Appl. Mater. Interfaces.

[2] Huang J., Lin J., Hsueh Y. (2016). Properties improvement of flexible silver nanowires transparent conductive thin film by using atmospheric plasma post-treatment. Nanosci. Nanotechnol. Lett..

[3] Han J.H., Kim D.H., Jeong E.G., Lee T.W., Lee M.K., Park J.W., Choi H.L.K.C. (2017). Highly conductive transparent and flexible electrodes including double stacked thin metal films for transparent flexible electronics. ACS Appl. Mater. Interfaces.

[4] Zhang B., Liu D., Liang Y., Zhang D., Yan H., Zhang Y. (2017). Flexible transparent and conductive films of reduced-graphene-oxide wrapped silver nanowires. Mater. Lett..

[5] Celle C., Mayousse C., Moreau E., Basti H., Carella A., Simonato J. (2012). Highly flexible transparent film heaters based on random networks of silver nanowires. Nano Res..

[6] Ding X., Yan J., Li T., Zhang L. (2012). Transparent conductive ITO/Cu/ITO films prepared on flexible substrates at room temperature. Appl. Surf. Sci..

[7] Choo D.C., Lee J.G., Kim T.W. (2015). Transparent conducting silver-nanowire-embedded poly(methyl methacrylate) nanocomposite films formed by using a transfer method. J. Nanosci. Nanotechnol..

[8] Hong S., Yeo J., Lee J., Lee H., Lee P., Lee S.S., Ko S.H. (2015). Selective laser direct patterning of silver nanowire percolation network transparent conductor for capacitive touch panel. J. Nanosci. Nanotechnol..

[9] Jiu J., Sugahara T., Ogo M.N., Araki T., Suganuma K., Uchida H., Shinozaki K. (2013). High-intensity pulse light sintering of silver nanowire transparent films on polymer substrates: The effect of the thermal properties of substrates on the performance of silver films. Nanoscale.

[10] Tokuno T., Nogi M., Karakawa M., Jiu J., Nge T.T., Aso Y., Suganuma K. (2011). Fabrication of silver nanowire transparent electrodes at room temperature. Nano Res..

[11] Jiang Y., Liu X., Li J., Zhou L., Yang X., Huang Y. (2015). Enhanced electrochemical oxidation of p-nitrophenol using single-walled carbon nanotubes/silver nanowires hybrids modified electrodes. J. Nanosci. Nanotechnol..

[12] Patel D.B., Patel M., Chauhan K.R., Kim J., Oh M.S., Kim J.-W. (2018). High-performing flexible and transparent photodetector by using silver nanowire-networks. Mater. Res. Bull..

[13] Wang J., Jiu J., Araki T., Nogi M., Sugahara T., Nagao S., Koga H., He P., Suganuma K. (2015). Silver nanowire electrodes: Conductivity improvement without post-treatment and application in capacitive pressure sensors. Nano Micro Lett..

[14] Kim S., Jeon H.R., An C.-H., An B.-S., Yang C.-W., Lee H.-J., Weon B.M. (2017). Improvement of conductivity of Ag nanowires-networked film using 1,8-diazabicyclo[5,4,0]undec-7-ene (DBU). Mater. Lett..

[15] Lee S.H., Lim S., Kim H. (2015). Smooth-surface silver nanowire electrode with high conductivity and transparency on functional layer coated flexible film. Thin Solid Films.

[16] Lim J.W., Cho D.Y., Eun K., Choa S.H., Na S.I., Kim J., Kim H.K. (2012). Mechanical integrity of flexible Ag nanowire network electrodes coated on colorless PI substrates for flexible organic solar cells. Sol. Energy Mater. Sol. Cells.

[17] Gupta R., Rao K.D.M., Srivastava K., Kumar A., Kiruthika S., Kulkarni G.U. (2014). Spray coating of crack templates for the fabrication of transparent conductors and heaters on flat and curved surfaces. ACS Appl. Mater. Interfaces.

[18] Sachse C., Meskamp L.M., Bormann L., Kim Y.H., Lehnert F., Philipp A., Beyer B., Leo K. (2013). Transparent, dip-coated silver nanowire electrodes for small molecule organic solar cells. Org. Electron..

[19] Madaria A.R., Kumar A., Ishikawa F.N., Zhou C. (2010). Uniform, highly conductive, and patterned transparent films of a percolating silver nanowire network on rigid and flexible substrates using a dry transfer technique. Nano Res..

[20] Scardaci V., Coull R., Lyons P.E., Rickard D., Coleman J.N. (2011). Spray deposition of highly transparent, low-resistance networks of silver nanowires over large areas. Small.

[21] De S., Higgins T.M., Lyons P.E., Doherty E.M., Nirmalraj P.N., Blau W.J., Boland J.J., Coleman J.N. (2009). Silver nanowire networks as flexible, transparent, conducting films: Extremely high DC to optical conductivity ratios. ACS Nano.

[22] Jing M.X., Li M., Chen C., Wang Z., Shen X. (2015). Highly bendable, transparent, and conductive AgNWs-PET films fabricated via transfer-printing and second pressing technique. J. Mater. Sci..

[23] Kim Y., Lee E., Lee J., Hwang D., Choi W., Kim J. (2016). High-performance flexible transparent electrode films based on silver nanowire-PEDOT: PSS hybrid-gels. RSC Adv..

[24] Jiang Y., Xi J., Wu Z., Dong H., Zhao Z., Jiao B., Hou X. (2015). Highly transparent, conductive, flexible resin films embedded with silver nanowires. Langmuir.

[25] Tian H., Xie D., Yang Y., Ren T.L., Lin Y.X. (2011). Flexible, ultrathin, and transparent sound-emitting devices using silver nanowires film. Appl. Phys. Lett..

[26] Min K., Umar M., Don H.S.H.Y.K., Heonsu J., Soonil L., Sunghwan K. (2017). Biocompatible, optically transparent, patterned, and flexible electrodes and radio-frequency antennas prepared from silk protein and silver nanowire networks. RSC Adv..

[27] Wang Y.H., Li Z.L., Hao A., Xie H., Li J.Z. (2017). Silver nanowires buried at the surface of mixed cellulose Ester as transparent conducting electrode. J. Nanosci. Nanotechnol..

[28] Li Z.L., Xie H., Jun D., Wang Y.H., Wang X.Y., Li J.Z. (2015). A comprehensive study of transparent conductive silver nanowires films with mixed cellulose ester as matrix. J. Mater. Sci. Mater. Electron..

